# Supplementary material for: Predicting cell population-specific gene expression from genomic sequence
Source: Front Bioinform. 2024 Mar 4;4:1347276. doi: 10.3389/fbinf.2024.1347276 (PMC10944912; doi:10.3389/fbinf.2024.1347276)
Supplement: Supplementary file 1 [file DataSheet1.PDF]

Supplementary data for

Predicting cell population-specific gene expression from genomic sequence

Lieke Michielsen[1,2,3], Marcel J.T. Reinders[1,2,3], Ahmed Mahfouz[1,2,3,*]

[1] Department of Human Genetics, Leiden University Medical Center, Leiden, 2333ZC, The Netherlands
[2] Leiden Computational Biology Center, Leiden University Medical Center, Leiden, 2333ZC, The Netherlands
[3] Delft Bioinformatics Lab, Delft University of Technology, Delft, 2628XE, The Netherlands

* Corresponding author: Ahmed Mahfouz (a.mahfouz@lumc.nl)

Supplementary figures

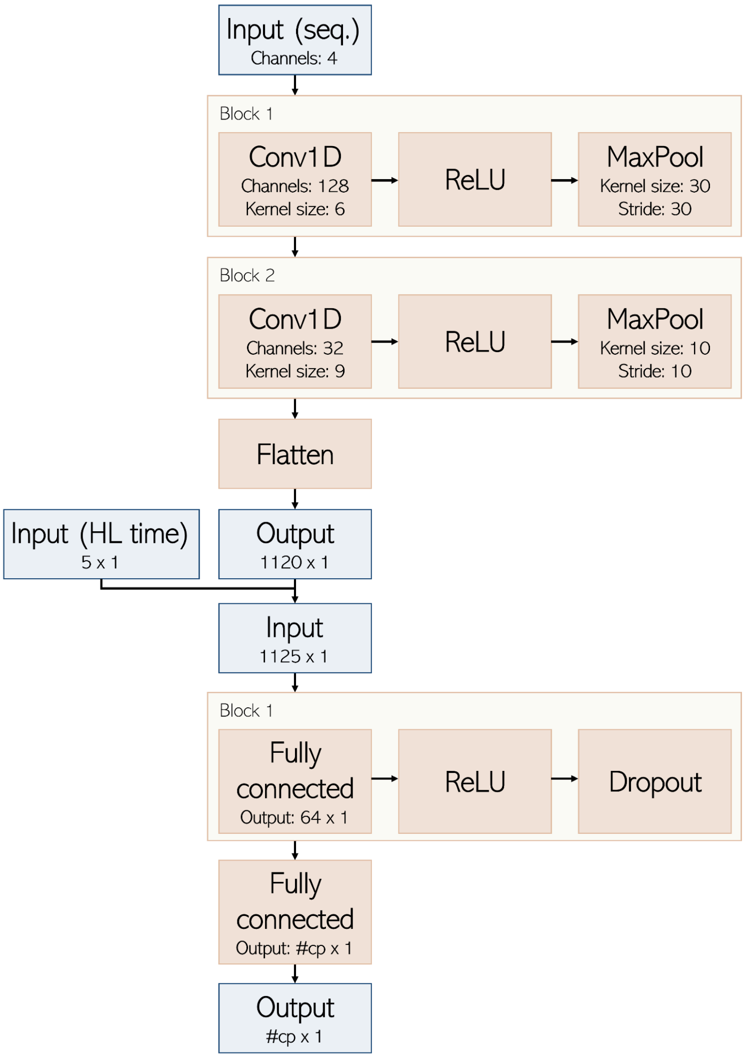

Figure S1 Detailed overview of scXpresso. The orange blocks indicate the convolutional blocks and fully connected layers. The blue blocks indicate the input and output of the different parts.

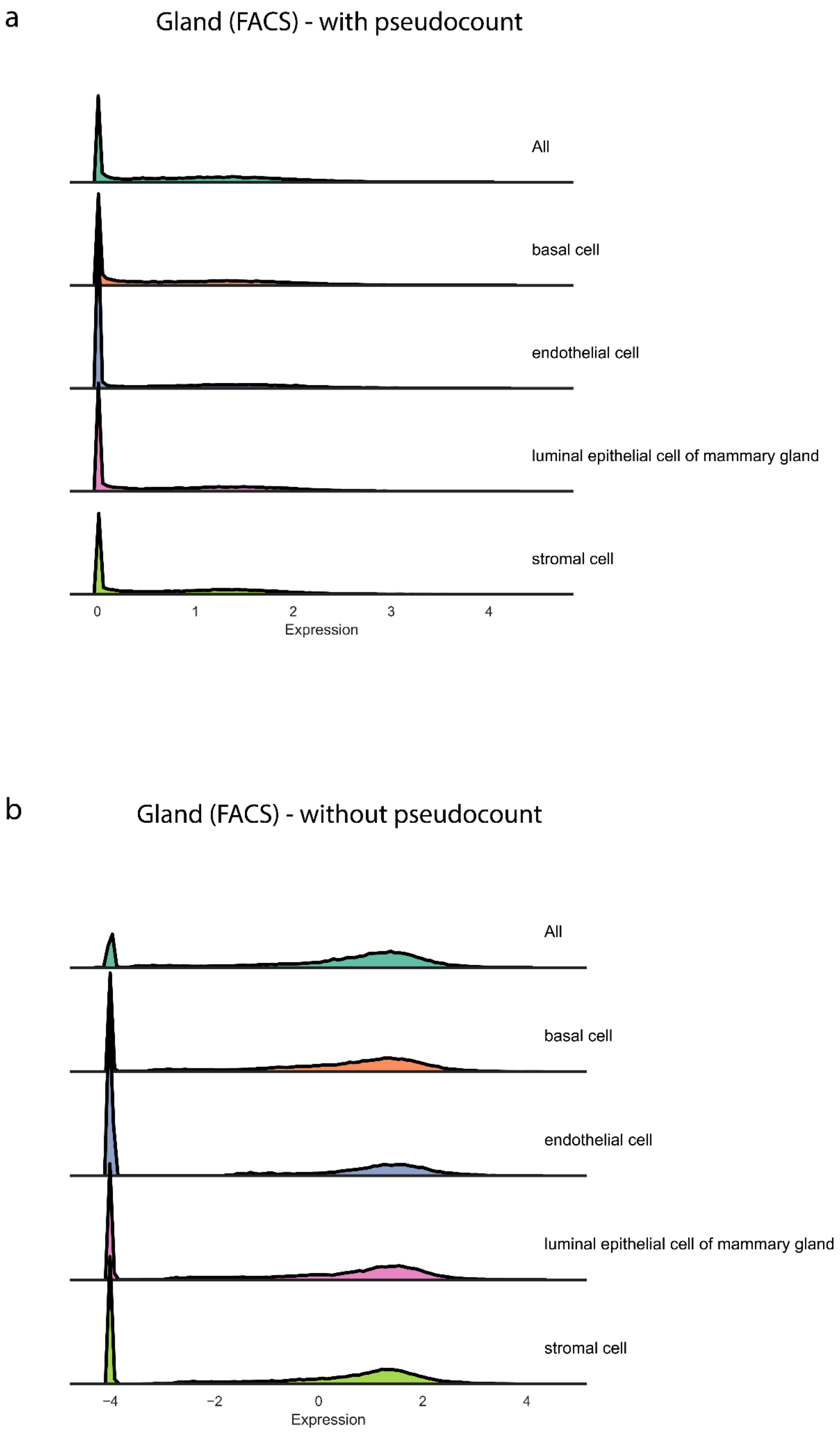

Figure S2 Distribution of the pseudobulk expression when we **a)** add or **b)** do not add the pseudocount.

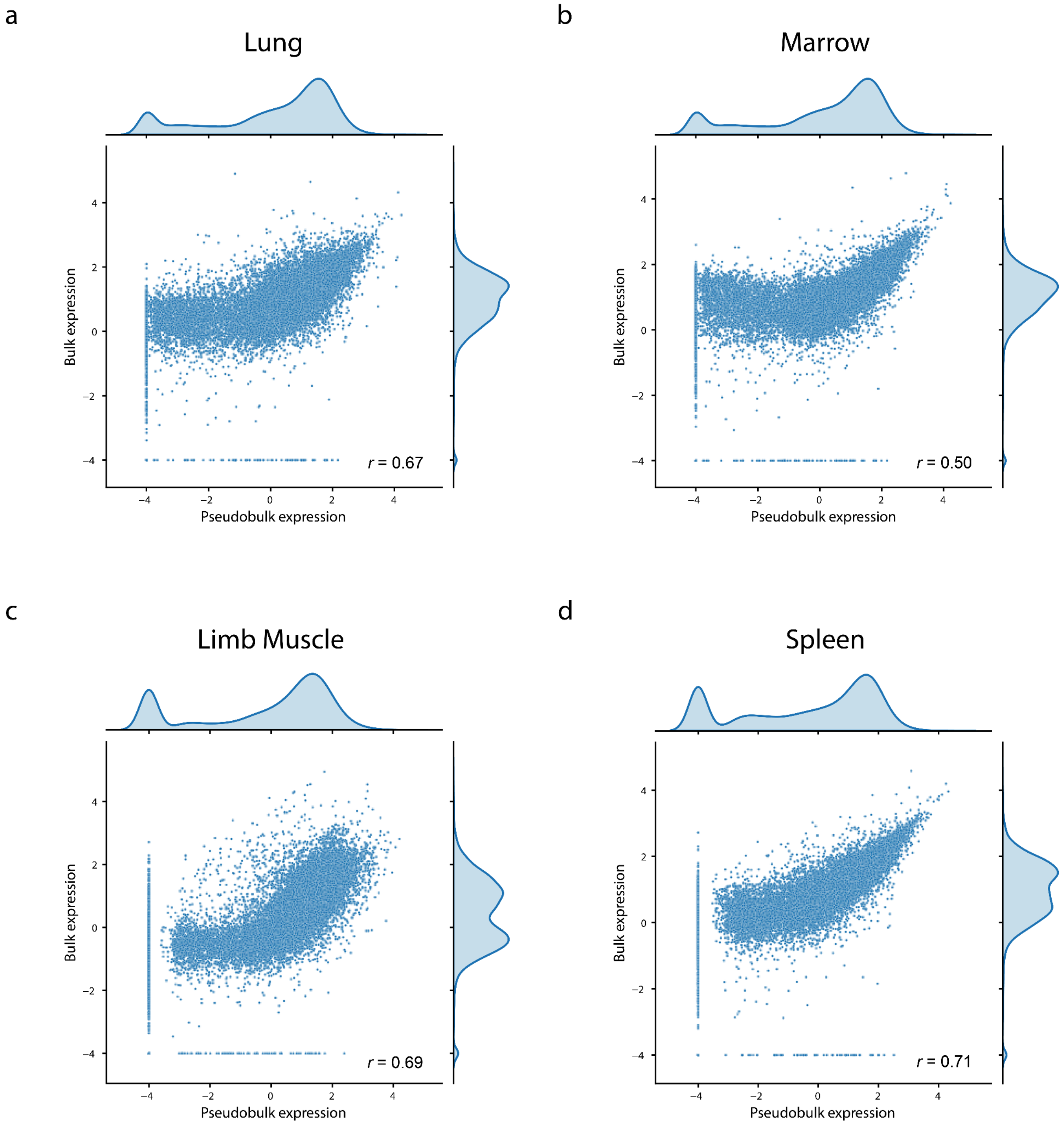

Figure S3 Pearson correlation between single-cell (FACS-sorted Smart-seq2) pseudobulk and bulk expression values for the four different tissues: **a)** Lung, **b)** Marrow, **c)** Limb Muscle, and **d)** Spleen.

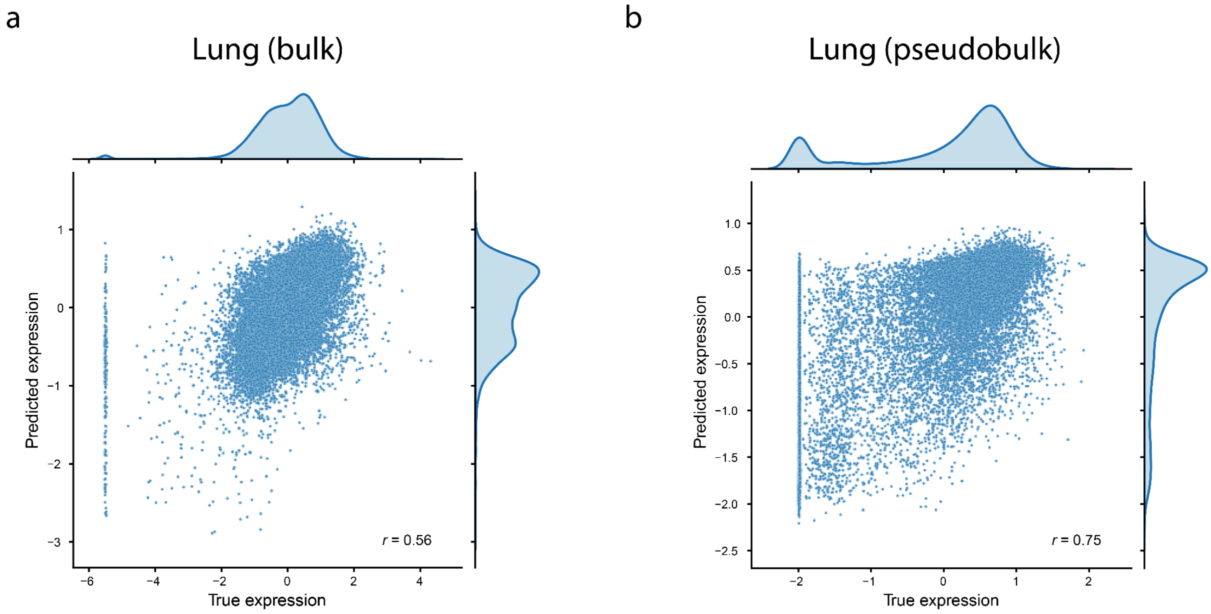

Figure S4 Scatterplots showing the models' performance on individual tissues (lung). **a)** Performance of scXpresso$_{t,b}$ on the lung bulk dataset. **b)** Performance of scXpresso$_{t,pb}$ on the lung pseudobulk dataset.

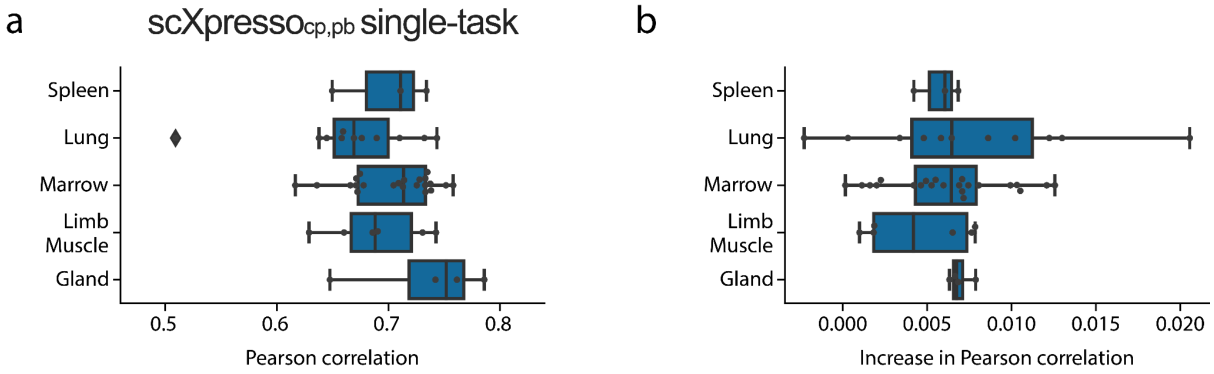

Figure S5 Comparing the performance of the scXpresso$_{cp,pb}$ single-task, and multitask models. **a)** Pearson correlation of the scXpresso$_{cp,pb}$ single-task models. **b)** Increased performance when comparing the multitask model to the single-task models. Every dot represents the **a)** performance or **b)** increase in performance of the model on a cell population in that tissue (median Pearson correlation across the 20 folds).

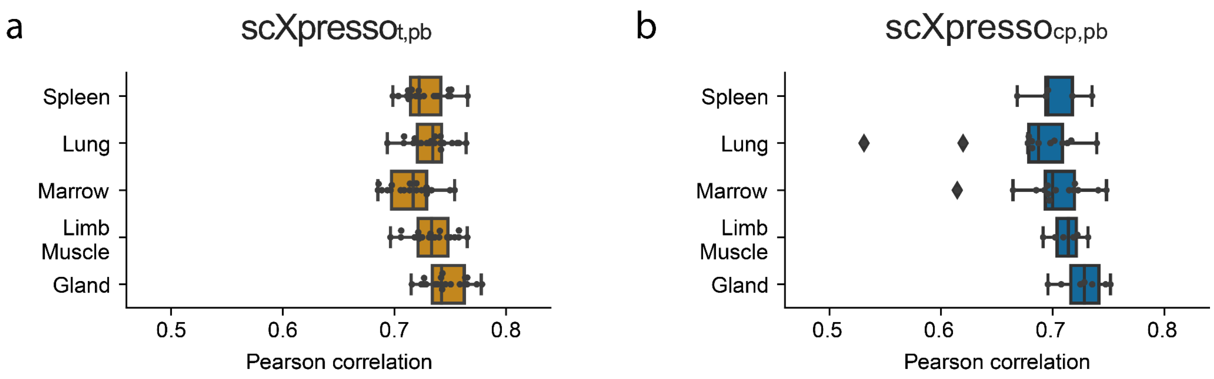

Figure S6 Pearson correlation of **a)** scXpresso$_{t,pb}$, and **b)** scXpresso$_{cp,pb}$ on the droplet scRNA-seq datasets. For scXpresso$_{t,pb}$ every dot is the performance across one fold of the 20-fold CV. For scXpresso$_{cp,pb}$, we summarized the performance per tissue, so every dot represents the performance of the model on a cell population in that tissue (median Pearson correlation across the 20 folds).

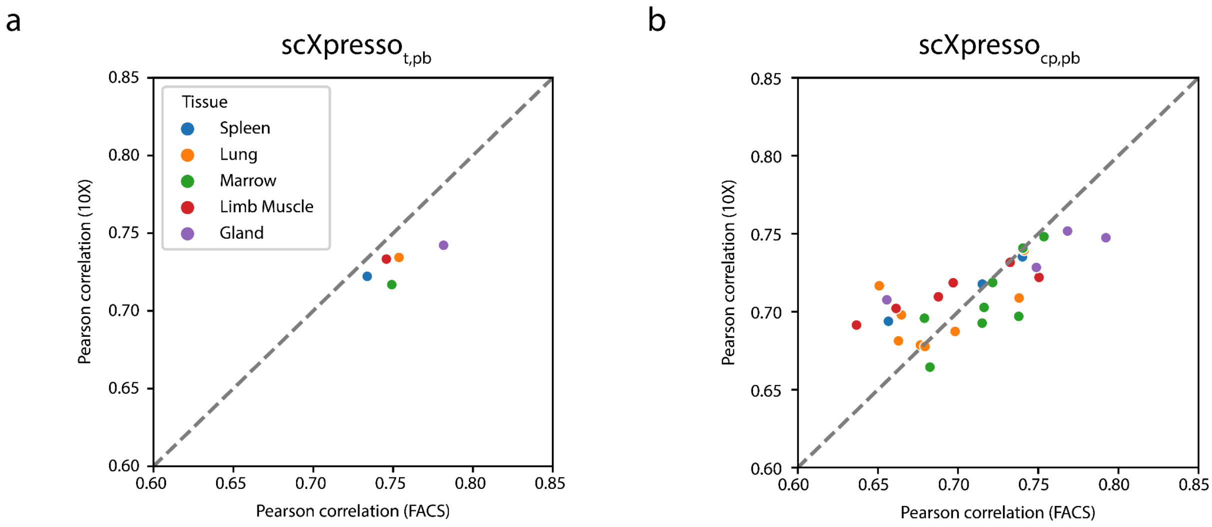

Figure S7 Comparing the performance of **a)** scXpresso$_{t,pb}$ and **b)** scXpresso$_{cp,pb}$ on FACS-sorted Smart-seq2 and 10X Genomics data. Every point represents the median Pearson correlation across the 20 folds on a tissue (scXpresso$_{t,pb}$) or a cell population (scXpresso$_{cp,pb}$). The points' color represents the tissue of origin. In **b)** only the cell populations defined in both protocols are shown.

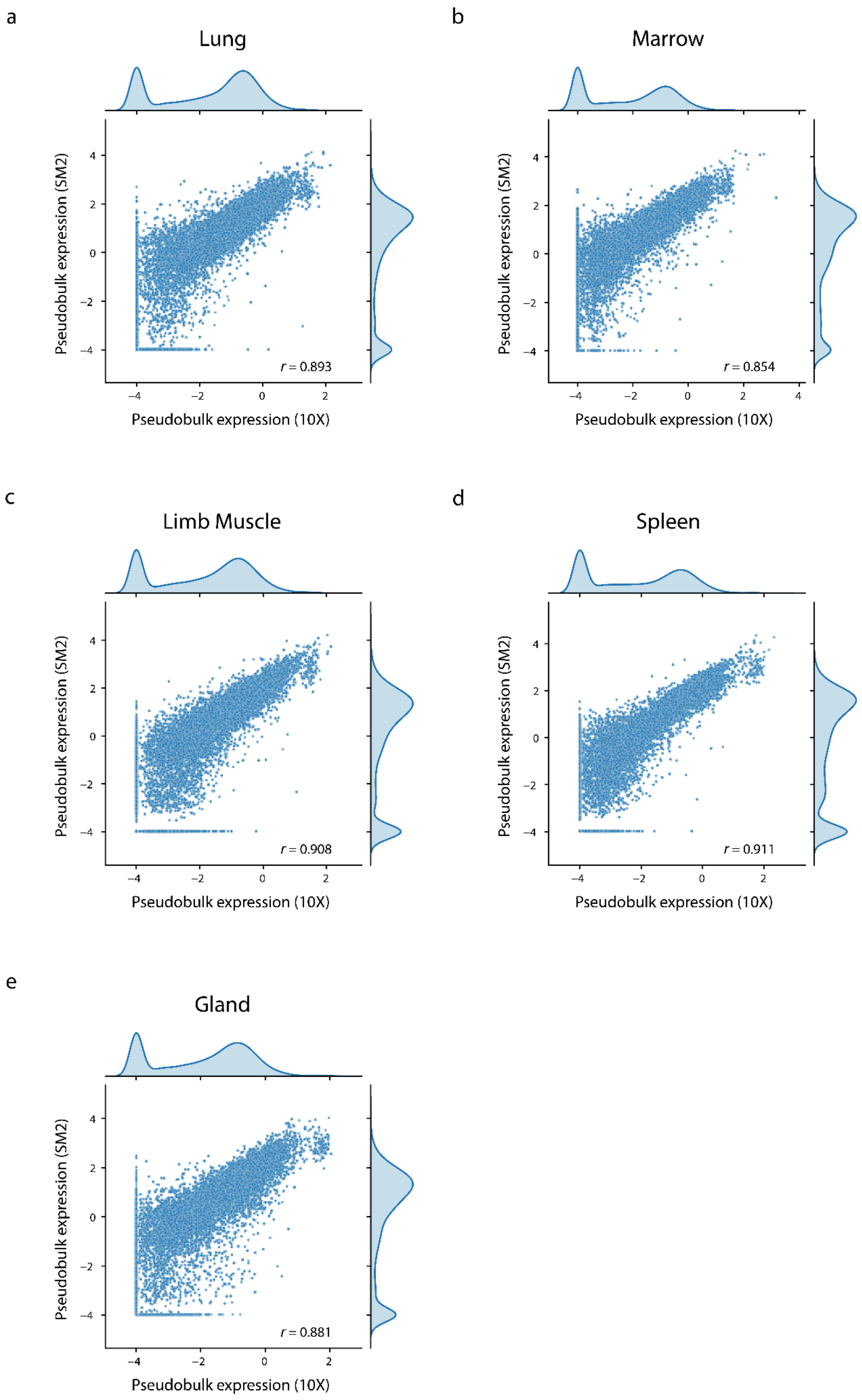

Figure S8 Pearson correlation between 10X Genomics and FACS-sorted Smart-seq2 pseudobulk expression values for the five different tissues: **a)** Lung, **b)** Marrow, **c)** Limb Muscle, **d)** Spleen, and **e)** Gland.

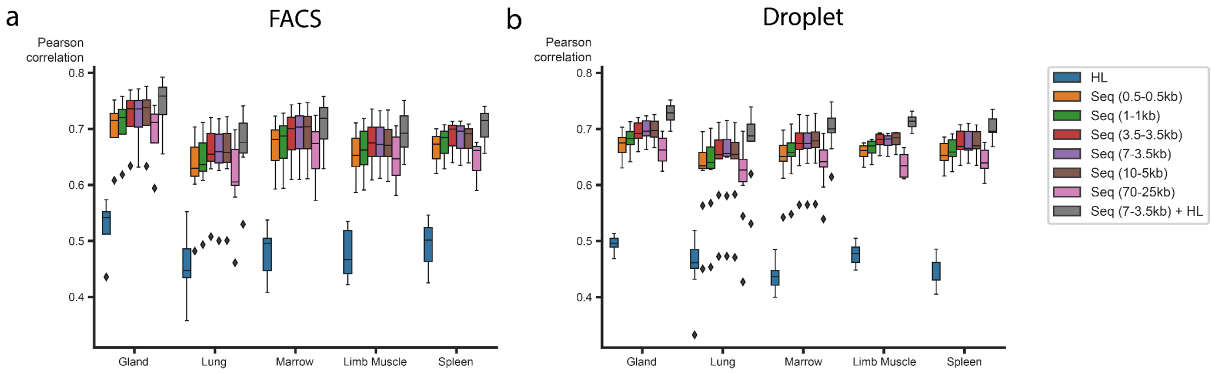

Figure S9 Comparison of different input features for scXpresso$_{cp,pb}$. Performance of scXpresso$_{cp,pb}$ on the **a)** FACS Smart-seq 2 and **b)** 10X droplet datasets from the Tabula Muris. Points in the boxplot represent the model's performance on a cell population in that tissue (medium Pearson correlation across the 20 folds).

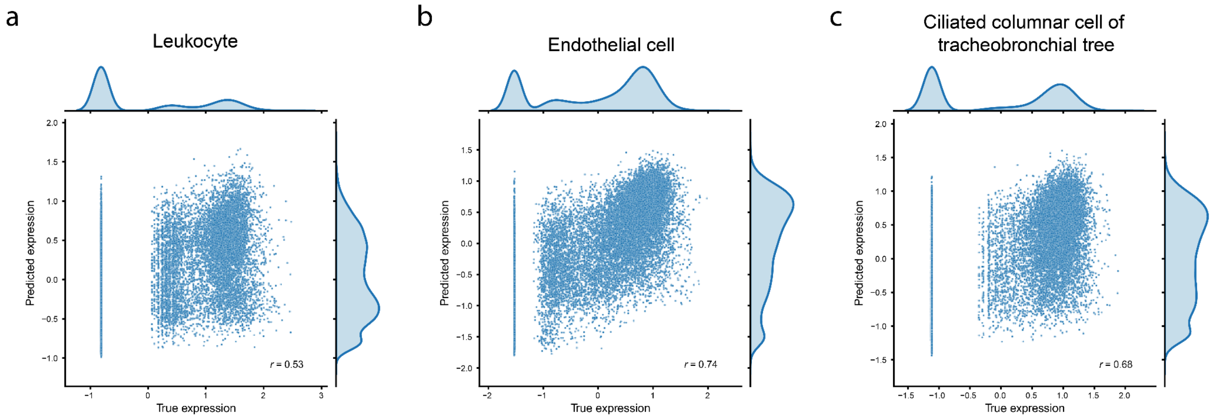

Figure S10 Performance of scXpresso$_{cp,pb}$ on the **a)** leukocytes, **b)** lung endothelial cells, and **c)** ciliated columnar cells. The leukocytes & ciliated cells are both very small populations (35 and 25 cells respectively), but more genes are non-zero in the ciliated cells (8,678 and 11,717 non-zero genes respectively).

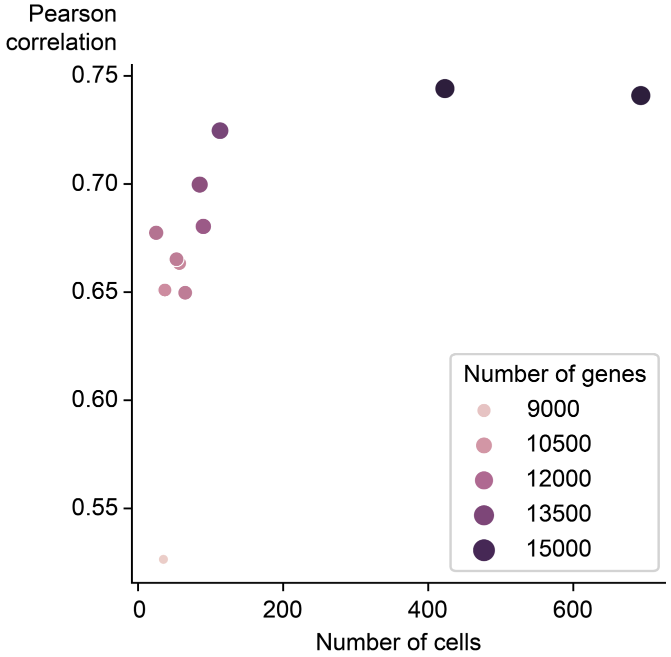

Figure S11 Performance of scXpresso$_{cp,pb}$ on the cell populations in the lung dataset versus the number of cells per cell population. Every dot is the median Pearson correlation across the 20-folds on that cell population. The dots are colored and sized according to the number of non-zero genes in the cell population.

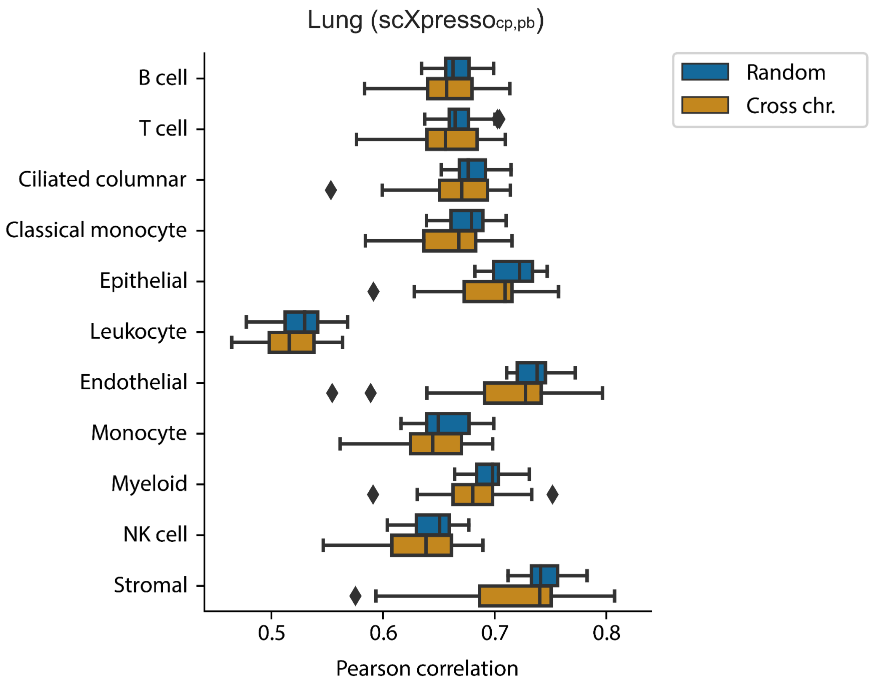

Figure S12 Performance of scXpresso$_{cp,pb}$ on the different lung cell populations. Every box shows the performance across the different folds of the cross-validation. The colors of the boxes indicate which cross-validation setup (random or cross-chromosomal) was used.

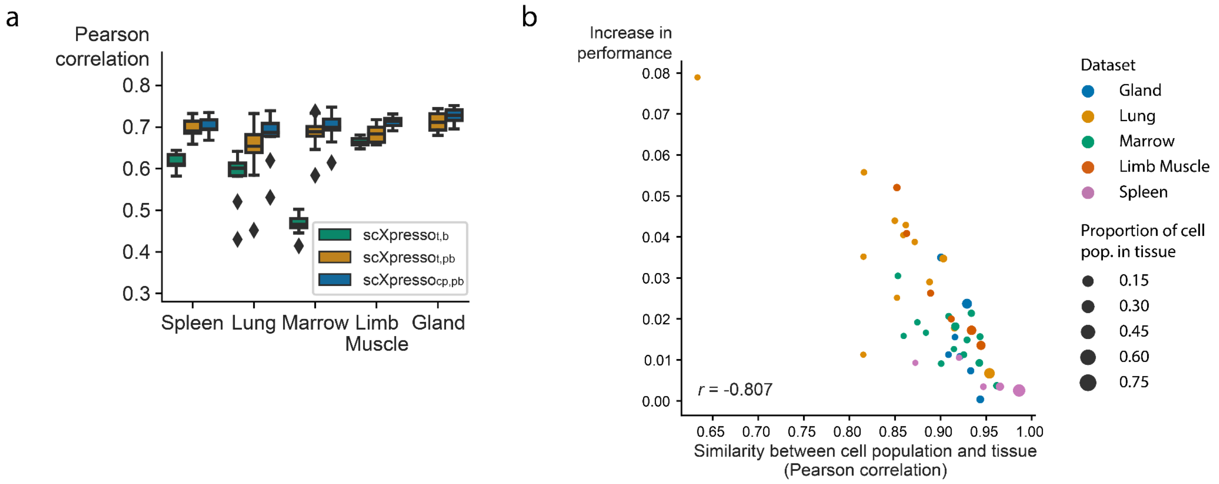

Figure S13 Performance of scXpresso on the 10X droplet Tabula Muris datasets. Figure 2A explains the setup of the experiment. **a)** Boxplot showing the Pearson correlation of the three scXpresso models on the cell population-specific task. Every point in the boxplot is the model's performance on one cell population in that tissue (median Pearson correlation across the 20 folds). **b)** Similarity between a cell population and corresponding tissue (Pearson correlation between the pseudobulk expression values) vs. the increase in performance.

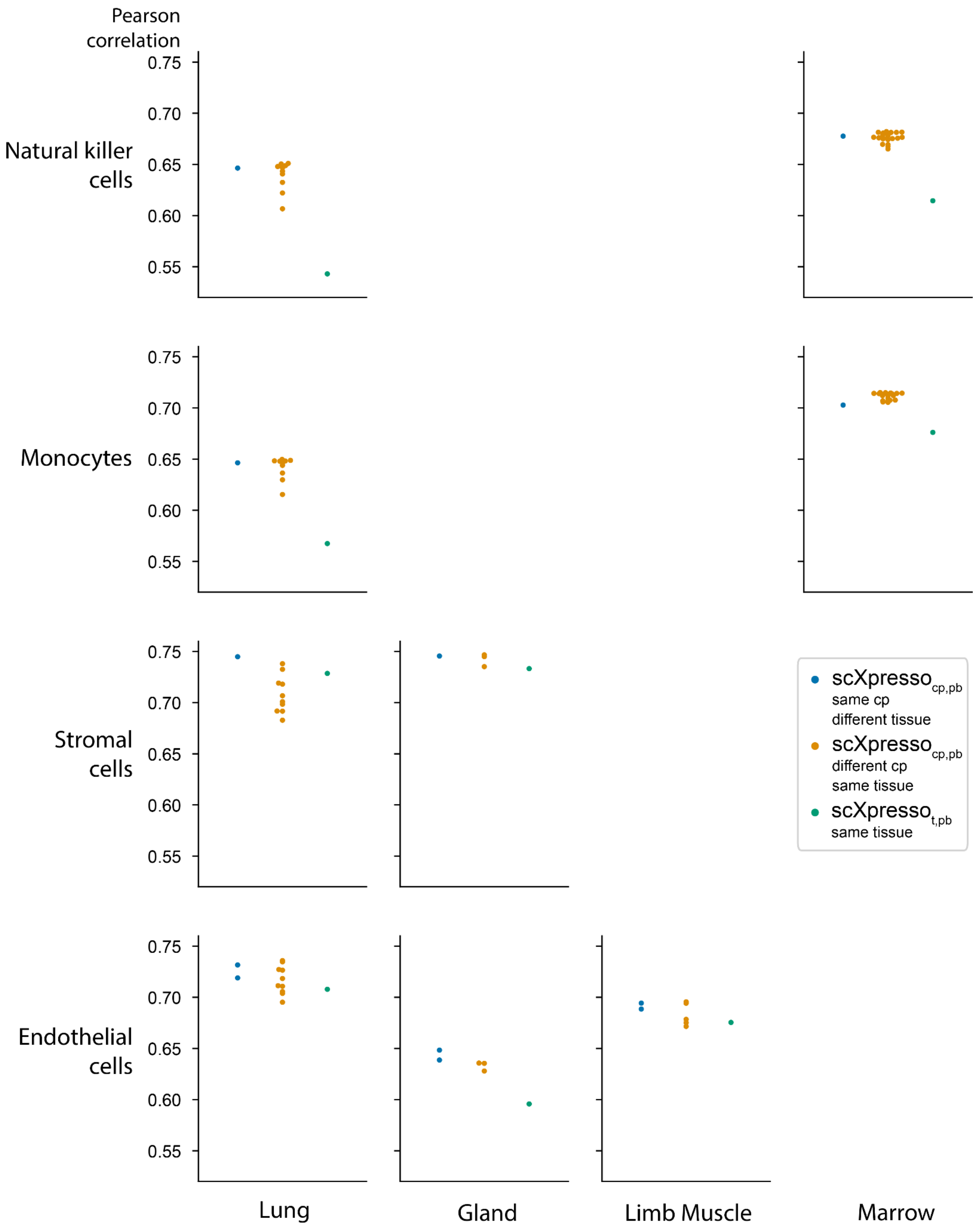

Figure S14 Performance (Pearson correlation) of three different types of models on different cell populations (rows) in different tissues (columns). Every dot is the median correlation of one model across the 20 folds. The datasets used here are the Tabula Muris FACS-sorted datasets. Some panels are empty since not all cell populations are defined in all tissues.

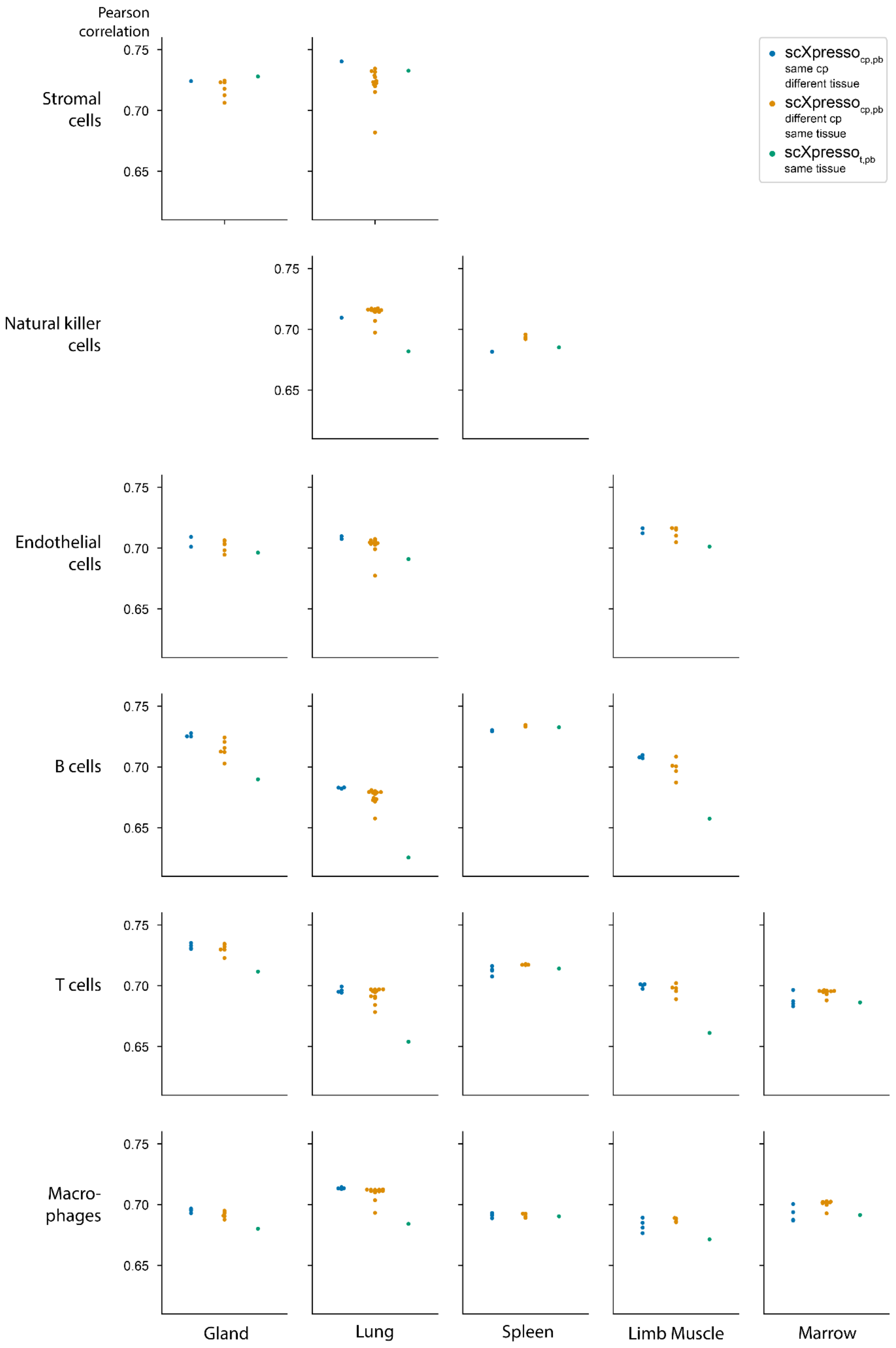

Figure S15 Performance (Pearson correlation) of three different types of models on different cell populations (rows) in different tissues (columns). Every dot is the median correlation of one model across the 20 folds. The datasets used here are the Tabula Muris droplet datasets. Some panels are empty since not all cell populations are defined in all tissues.

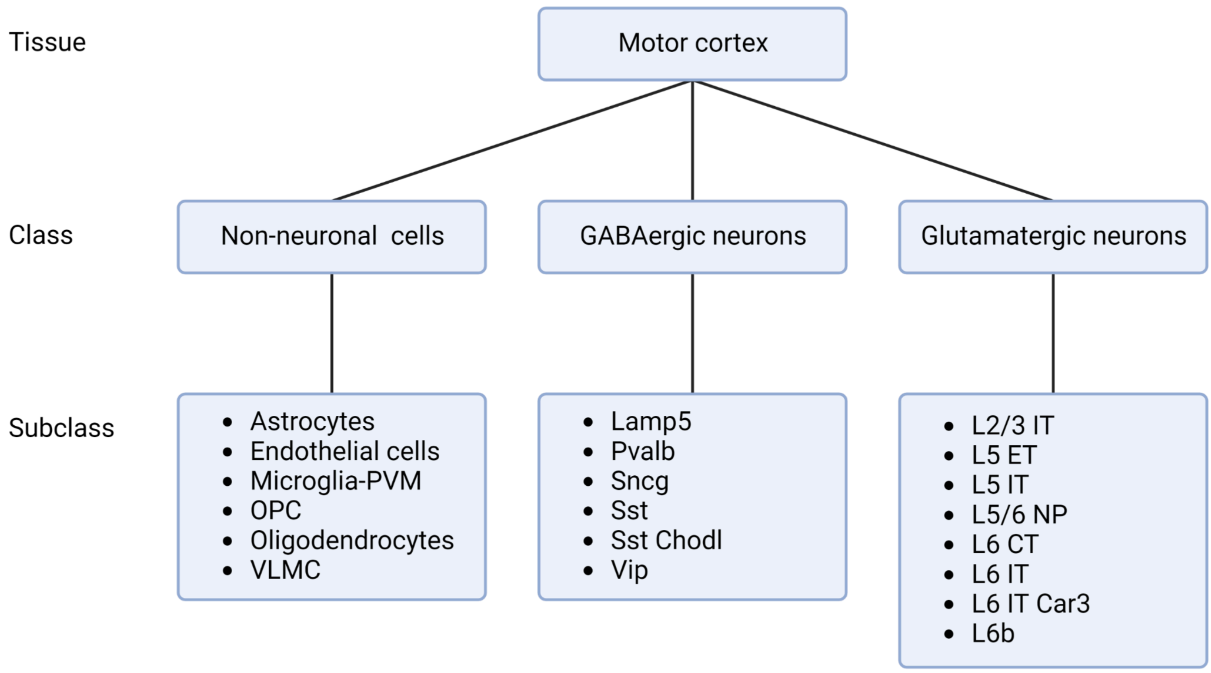

Figure S16 Hierarchical representation of the classes and subclasses in the motor cortex.

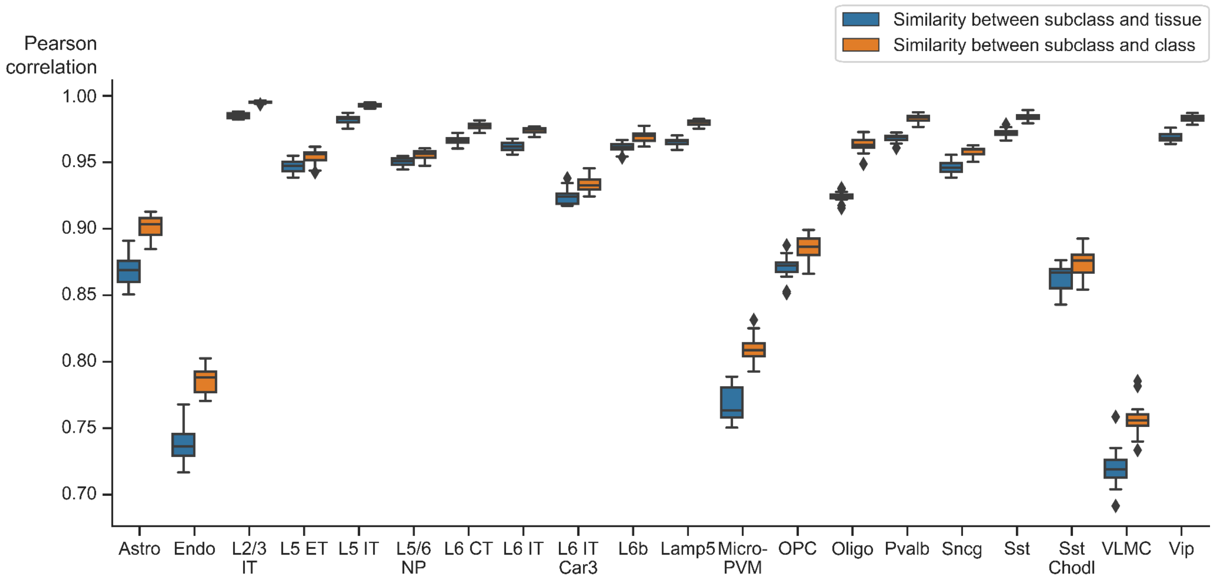

Figure S17 Similarity between the true expression values of a subclass and the corresponding class and tissue (Pearson correlation). Every dot in the boxplot is the similarity across one fold of the 20-fold CV.

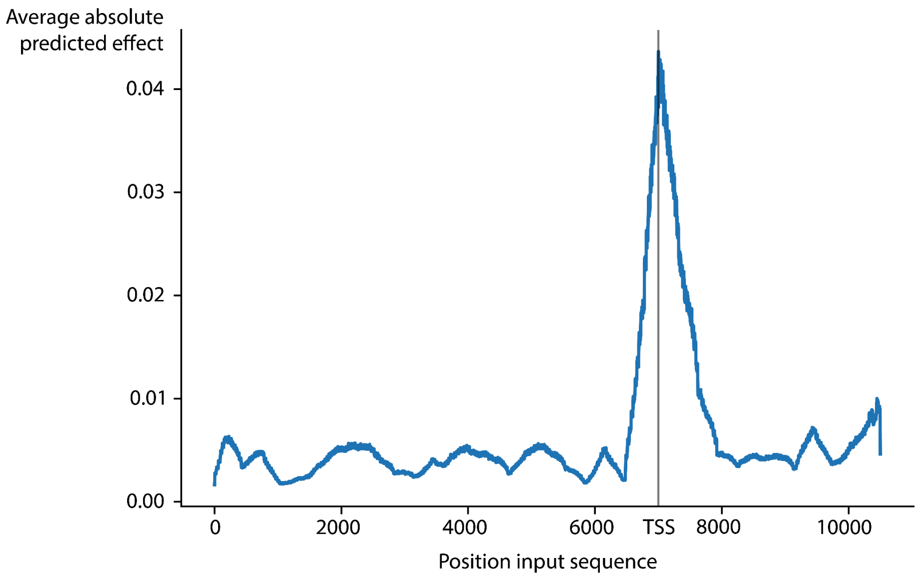

Figure S18 Average absolute predicted effect for different positions in the input sequence.

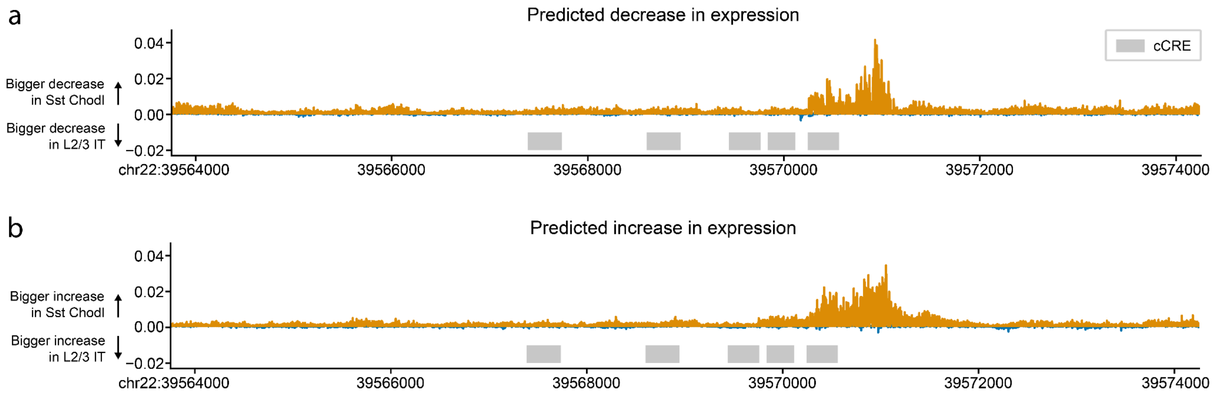

Figure S19 Difference between the *in-silico* mutagenesis profiles of Sst Chodl and L2/3 IT subclass. In **a)** both subclasses are predicted to decrease in expression and in **b)** both to decrease in expression. Positive values (plotted in orange) mean that a bigger effect is predicted for Sst Chodl cells, while negative values (plotted in blue) mean that a bigger effect is predicted for L2/3 IT. The grey rectangles indicate the position of candidate cis-Regulatory Elements (cCREs) derived from ENCODE data.

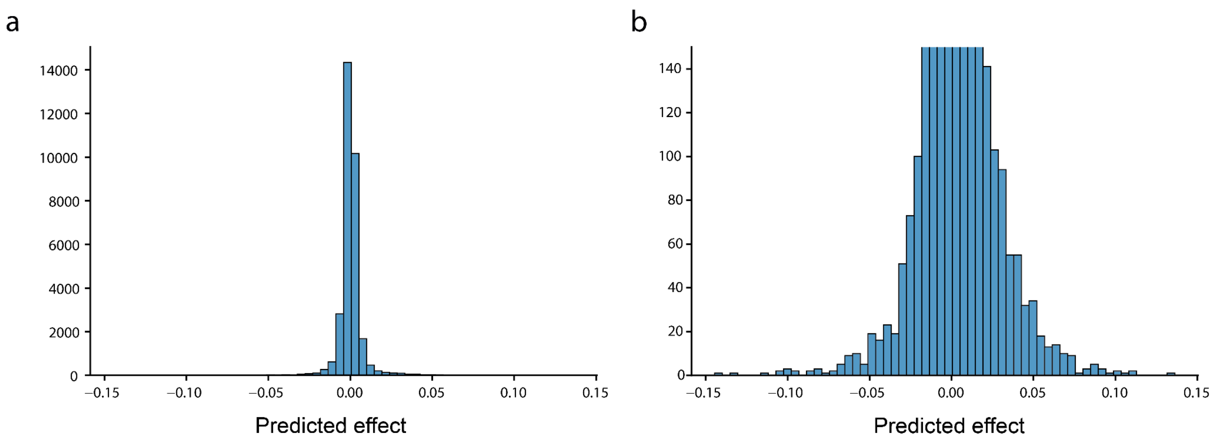

Figure S20 Histograms showing **a)** the complete distribution and **b)** the focussed version of the predicted effect of all mutations (n = 31,500) for *CACNA1I* in the Sst Chodl subclass.

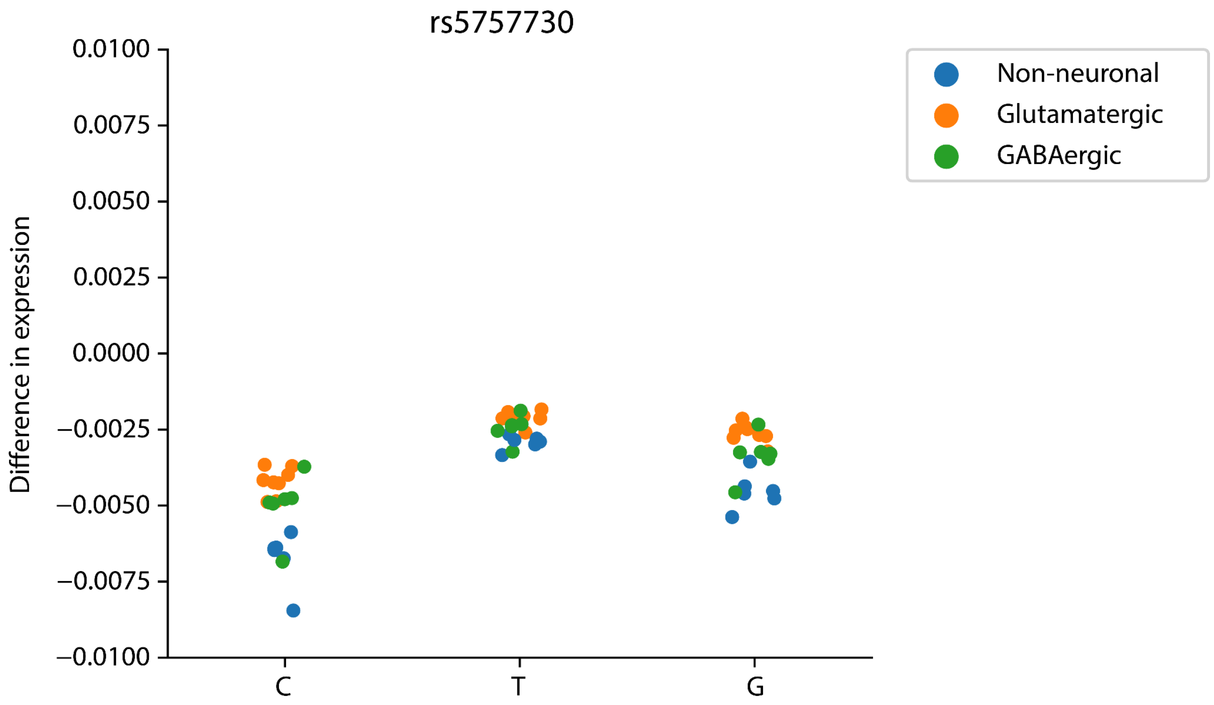

Figure S21 The predicted effect of the three substitutions for rs5757730 on *CACNA1I* expression. Every dot is one subclass and the dots are colored according to the class. Note the scale on the y-axis. The predicted effect is very small.

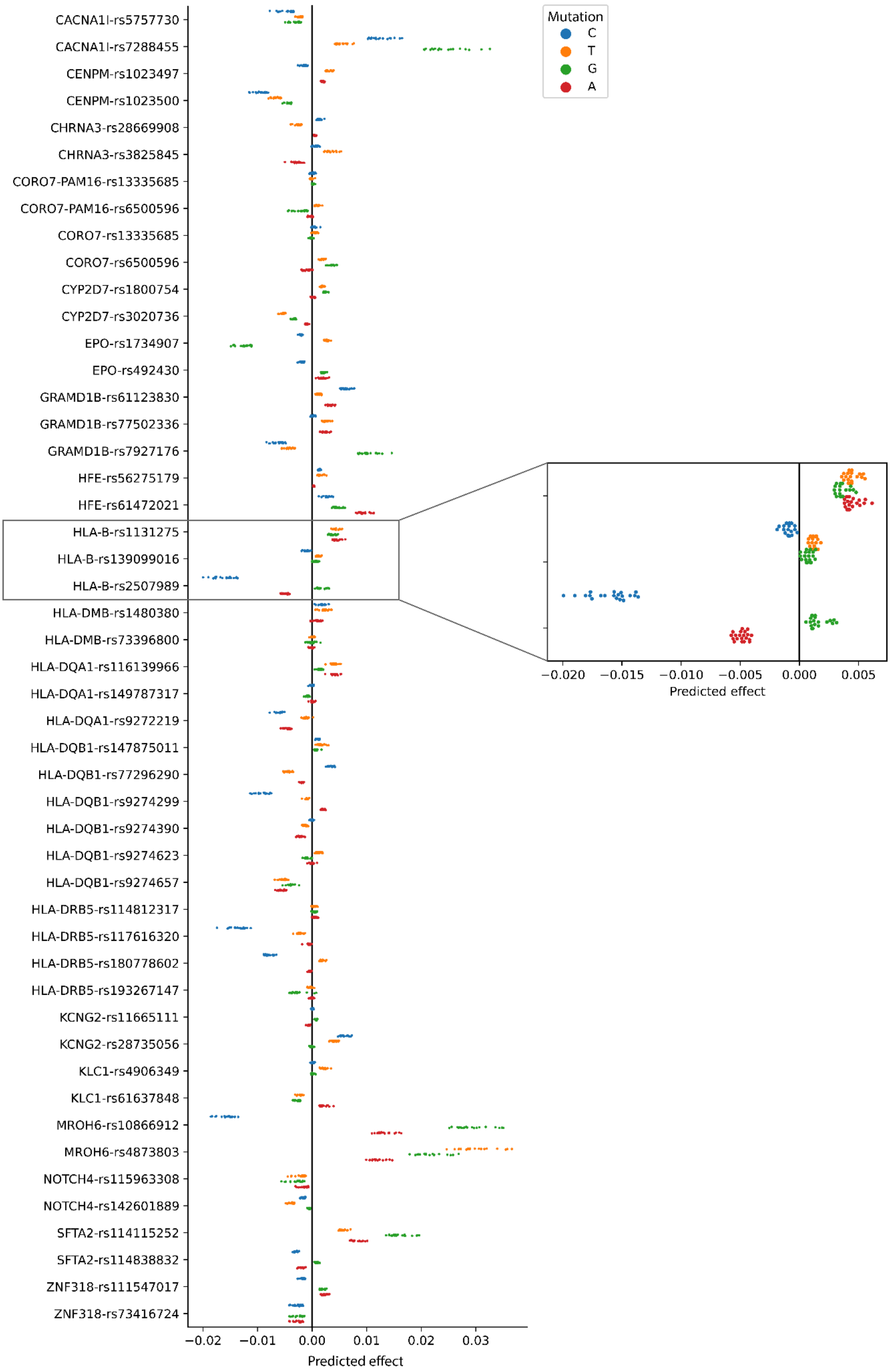

Figure S22 We show the predicted effect for variants that are related to schizophrenia. For every variant, we indicate for which gene we predicted the effect. For every variant, you see three different substitutions (three colors). Every dot represents the predicted effect for one cell population.

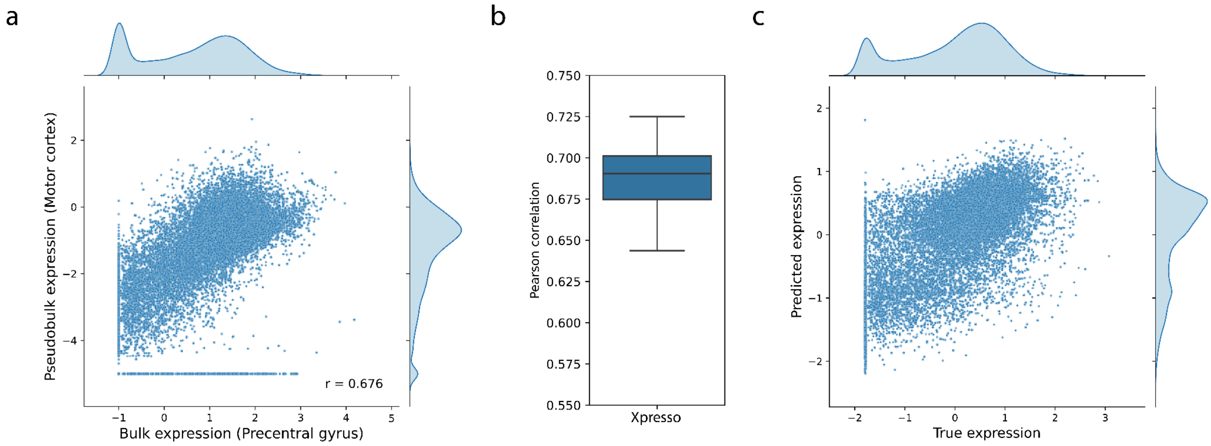

Figure S23 Performance of Xpresso on the precentral gyrus dataset. **a)** Correlation between the bulk values for the precentral gyrus and the pseudobulk values for the motor cortex. **b)** Performance of Xpresso on the precentral gyrus dataset across 20-fold cross-validation. **c)** Scatterplot showing the predictions of Xpresso.

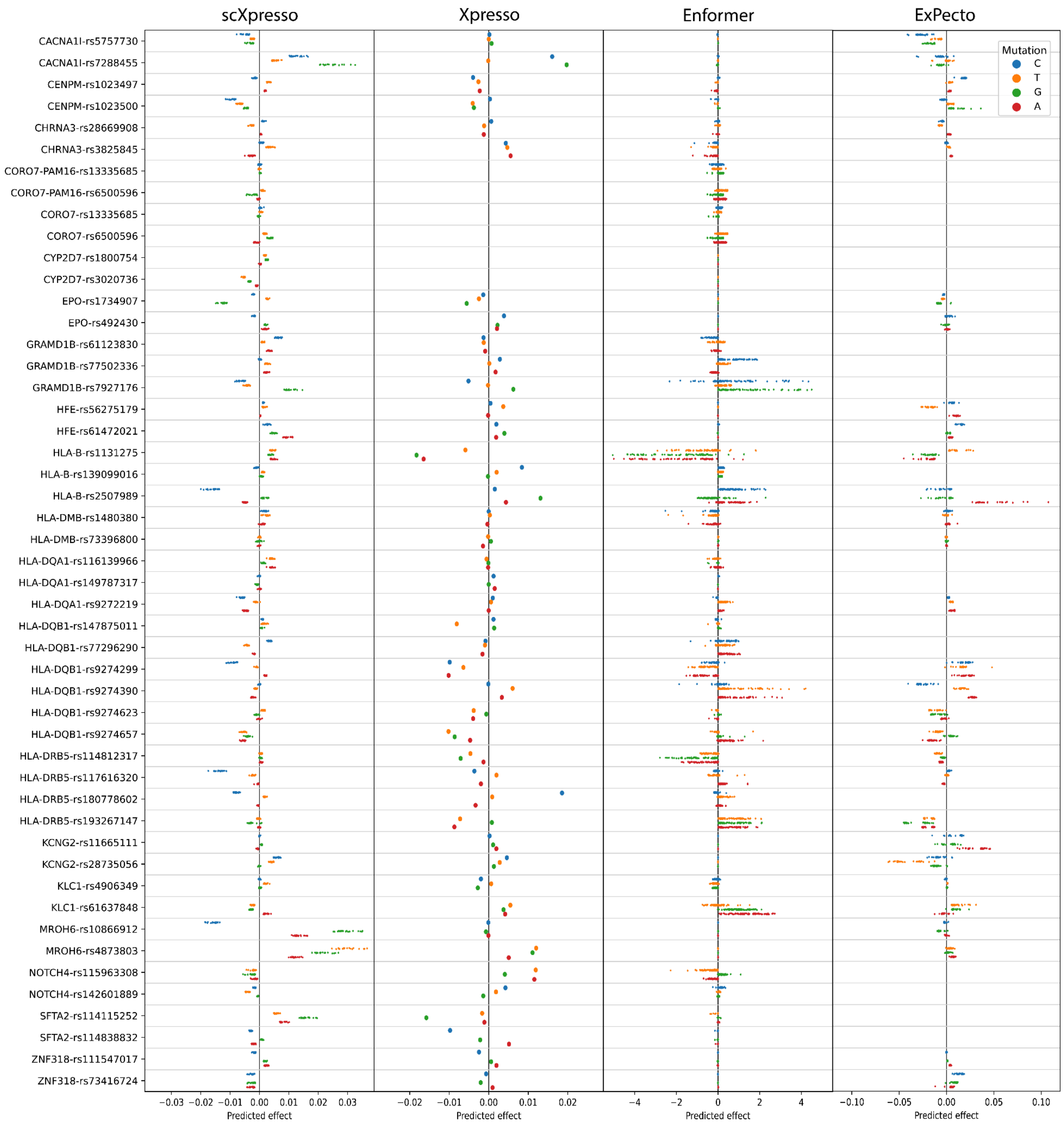

Figure S24 The predicted effect for variants related to schizophrenia. For every variant, we predicted the effect using scXpresso, Xpresso, Enformer, and ExPecto. For scXpresso, every dot indicates the prediction of a subclass. For Enformer every dot indicates the prediction of a CAGE track related to the brain. For ExPecto every dot indicates the prediction on a certain brain tissue. For every variant, you see three different substitutions (three colors). Some variants are not predicted by all methods (see Table S3).

Supplementary Tables

Table S1: Coding scheme for nucleotides

	**A**	**C**	**T**	**G**	**M**	**W**	**R**	**Y**	**S**	**K**	**B**	**D**	**V**	**G**	**N**
**A**	1	0	0	0	1/2	1/2	1/2	0	0	0	0	1/3	1/3	1/3	1/4
**C**	0	1	0	0	1/2	0	0	1/2	1/2	0	1/3	0	1/3	1/3	1/4
**T**	0	0	1	0	0	1/2	0	1/2	0	1/2	1/3	1/3	0	1/3	1/4
**G**	0	0	0	1	0	0	1/2	0	1/2	1/2	1/3	1/3	1/3	0	1/4

Table S2: Performance of the Xpresso model using different input features

	**Input Features**	r^2
**Human**	Sequence	0.521
	8 HL time features	0.300
	Seq. + 8 HL	0.563
	Seq. + 5 HL	0.567
	Results reported in Xpresso paper	0.590
**Mouse**	Sequence	0.643
	8 HL time features	0.479
	Seq. + 8 HL	0.713
	Seq. + 5 HL	0.701
	Results reported in Xpresso paper	0.710

Table S3: List of CAGE tracks related to the brain in the Enformer model

See Supplementary file.

Table S4: List of ExPecto outputs related to the brain

See Supplementary file.

Table S5: List of variants related to Schizophrenia used

See Supplementary file.

Table S6: Overview of Tabula Muris datasets (single-cell) used during this study.

Tissue	Protocol	Genes (expressed)	Cells	Cell populations
Gland	10X	20467 (16039)	4481	7
	FACS	20467 (17582)	2663	4
Limb Muscle	10X	20467 (15809)	4543	6
	FACS	20467 (16742)	1151	6
Spleen	10X	20467 (14597)	9573	5
	FACS	20467 (16725)	1718	3
Lung	10X	20467 (15954)	5404	13
	FACS	20467 (17703)	1923	11
Bone marrow	10X	20467 (14682)	4112	14
	FACS	20467 (18742)	5355	22

Table S7: Overview of Tabula Muris datasets (bulk) used during this study.

Tissue	Genes (expressed)	Samples
Limb Muscle	20467 (20214)	54
Spleen	20467 (20258)	56
Lung	20467 (20282)	55
Bone marrow	20467 (20263)	54
